# Time Course of Severe Fever With Thrombocytopenia Syndrome Virus and Antibodies in Patients by Long-Term Follow-Up Study, China

**DOI:** 10.3389/fmicb.2021.744037

**Published:** 2021-10-12

**Authors:** Lifen Hu, Qinxiang Kong, Yanyan Liu, Jiajia Li, Tingting Bian, Xuejiao Ma, Ying Ye, Jiabin Li

**Affiliations:** ^1^Department of Infectious Diseases, The First Affiliated Hospital of Anhui Medical University, Hefei, China; ^2^Anhui Center for Surveillance of Bacterial Resistance, Hefei, China; ^3^Department of Infectious Diseases, Chaohu Hospital of Anhui Medical University, Hefei, China

**Keywords:** severe fever with thrombocytopenia syndrome virus, IgM antibody, IgG antibody, inflammatory factors, follow-up

## Abstract

**Objectives:** The objective was to describe the changes of severe fever with thrombocytopenia syndrome virus (SFTSV) and antibody in the disease course and explore the relationship between antibody titers and patients’ prognosis.

**Methods:** The levels of SFTSV, virus-specific immunoglobulin M (IgM), immunoglobulin G (IgG) titers, and cytokines in 37 patients with severe fever with thrombocytopenia syndrome (SFTS) were measured dynamically by real-time PCR and ELISA during the disease course; IgG titers were followed up in 53 cases. The correlation analysis of antibody titers with individual serum cytokines was calculated using the Spearman test.

**Results:** The average time of SFTSV duration in individual serum was 22.45 ± 7.6 days from onset. We found SFTSV turned negative within the 10th day from the onset in two patients. SFTSV-specific IgM seroconversion occurred as early as within 3 days from the onset, increased gradually within the first 2 months, decreased gradually 3 months later, and disappeared after 6 months in all the patients. The average time of SFTSV-specific IgG antibody seroconversion was at 17 days from onset in the patients; the time was later in severe cases than in mild cases (23 ± 1.4 vs. 14.3 ± 1.0 days, *p* < 0.0001). IgG titers were maintained at the peak levels during the periods from 6 months to 1 year and decreased from the second year gradually. Severe cases had higher IgG levels than mild cases and also had a slower decreasing trend. During follow-up, only one lost IgG antibody 7 years later; no chronic infection and sequela were found among the 53 patients. None of the patients had SFTSV reinfection even if they were bitten by ticks again. The correlation analysis showed a positive relationship between inflammatory factors and IgG antibody levels.

**Conclusion:** IgM antibody has important value in early diagnosis of SFTS. A moderate inflammatory response is beneficial for production and duration of IgG antibodies.

## Introduction

Severe fever with thrombocytopenia syndrome (SFTS) is an emerging infectious disease that was first identified in 2009 in China. The causative agent of SFTS is a novel phlebovirus in the Bunyaviridae family, which was named SFTS virus (SFTSV) (Yu et al., 2011). Since its initial discovery, SFTS has been epidemic in more than 20 provinces in China, which has threatened the health of Chinese people seriously (Guo et al., 2016; Zhan et al., 2017; Bopp et al., 2020; Li et al., 2021). Anhui province has shown a higher disease burden of SFTS (Zhan et al., 2017; Bopp et al., 2020; Li et al., 2021). Besides China, SFTSV infections have also been confirmed in Japan, South Korea, Vietnam, and Pakistan (Li, 2015; Tran et al., 2019; Bopp et al., 2020; Miao et al., 2020). The virus was not only transmitted by tick bites but although by person-to-person transmission (Gong et al., 2018; Jung et al., 2019). SFTSV-RNA gene mainly expresses nucleocapsid protein (N protein) which is the most immunogenic antigen; a specific antibody usually plays a key role in the antiviral process (Jiao et al., 2012). Specific immunoglobulin M (IgM) antibody to SFTSV has been reported to appear at a median of 9 days after disease onset, and lower IgM levels were measured in severe cases (Jung et al., 2019). The immune response has been confirmed to play an important role in the pathogenesis of SFTS (Sun et al., 2012a; Hu et al., 2018). For this emerging infectious disease, it remains unclear as to the existent duration of SFTS virus-specific immunoglobulin antibodies and whether the patients can be reinfected by SFTSV.

Limited information is available regarding the association of immune response with antibody duration and of antibody response with SFTSV reinfection. In this study, we performed a prospective study on SFTSV infected cases to determine the attribution of inflammatory factors levels to antibody duration. A long-term follow-up (longest at 8 years) study was also performed on post-infection patients to study the levels of specific IgG antibodies elicited by SFTSV and reinfection incidence of SFTS.

## Materials and Methods

### Study Population and Sample Collection

Fifty-three patients diagnosed with positive SFTSV in the First Affiliated Hospital of Anhui Medical University were included in this study from March 2011 to December 2018. Patients with rickettsial diseases, human granulocytic anaplasmosis, or underlying diseases including autoimmune diseases, hematological diseases, and other chronic disease were excluded. Fifty-three SFTS patients included 3 patients in 2011, 4 patients in 2012, 6 patients in 2013, 3 patients in 2014, 16 patients in 2015, 12 patients in 2016, 5 patients in 2017, and 4 patients in 2018. According to the Guidelines for the Prevention and Treatment of Severe Fever with Thrombocytopenia Syndrome (2010 Edition) (Ministry of Health of the People’s Republic of China, 2011), patients were classified as severe and mild disease groups. Among the 37 patients from 2015 to 2018, serum samples were collected at 3-day intervals during the hospitalization period. During the follow-up, serum samples were collected every 3 months in the first year and every 6 months in the patients 1 year after discharge in the 37 patients. Among the 16 patients from 2011 to 2014, serum samples were also collected every 6 months starting from 2015. The demographic data, clinical manifestations, and laboratory test results of patients were obtained. Written informed consent was provided by all patients, following the Declaration of Helsinki. The study and relevant experiments were approved by the local Ethics Committee of the First Affiliated Hospital of Anhui Medical University (No.Quick-PJ2021-08-31).

### Severe Fever With Thrombocytopenia Syndrome Virus RNA Detection

RNA was extracted from peripheral blood using a high-purity viral RNA kit (Qiagen) according to the manufacturer’s instructions. SFTSV was amplified using specific primers and probes by real-time reverse-transcription polymerase chain reaction (RT-PCR) under conditions previously described (Sun et al., 2012b). RT-PCR conditions were as follows: 50°C for 30 min, 95°C for 15 min, 40 cycles at 95°C for 15 s, and 55°C for 40 s. Amplification and detection were performed with an Applied Biosystems 7500 Real-time PCR system (Applied Biosystems, Foster City, CA). The standard curve was generated using different standard RNA concentrations ranging from 1.0 × 107 to 1.0 × 103 copies/μl obtained by 10-fold serial dilutions.

### Severe Fever With Thrombocytopenia Syndrome Virus Antibody Detection

Sera were tested for SFTSV N protein-specific IgM and IgG antibodies using an ELISA kit (Da An Gene Co. Ltd., Guangzhou, China). For initial screening, a 1:40 diluted serum sample was used to determine whether the sample was positive for viral antibodies. Positive serum samples were further diluted in a twofold serial dilution starting at 1:80 for the assay to obtain endpoint titers determined by the cutoff values set by positive and negative controls as provided with the ELISA kit.

### Measurement of Inflammatory Mediators

As described in our previous study (Hu et al., 2018), inflammatory mediators including interleukin (IL)-6, IL-10, IL-8, and tumor necrosis factor (TNF)-α were measured in SFTS patients at the acute stage of disease using MILLIPLEX^®^ MAP human cytokine/chemokine magnetic bead panel kits (Merck Millipore, Germany) according to the manufacturer’s instructions (Luminex^®^ 200^TM^ System, Life Technologies, Grand Island, NY). Inflammatory mediators including interleukin (IL)-6, IL-10, IL-8, and tumor necrosis factor (TNF)-α were measured in plasma samples from patients.

### Statistical Analyses

Data were analyzed using SPSS, version 20.0 (SPSS Inc., United States). Quantitative variables were expressed as means ± standard deviation (SD) or as medians (interquartile range); categorical variables were expressed as the number (percentage). Between the two groups of mild and severe cases, comparisons of Log10 [1/(IgG antibody titers)], IgM seroconversion, and IgG seroconversion time were calculated by unpaired *t*-test or non-parametric tests. Graphpad prism 5 software was used to compare values in groups and calculate the correlation coefficients and significance values of two variables. The correlation analysis of antibody titers with individual serum cytokines was calculated using the methods of the Pearson correlation coefficient. A two-sided *p*-value < 0.05 was considered statistically significant.

## Results

### Characteristics of Severe Fever With Thrombocytopenia Syndrome Cases Followed Up

According to the patients’ medical history, all the patients in this research were infected by SFTSV for the first time. Thirty-two (60%) patients were male and 21 (40%) were female among the 53 SFTS patients. All the patients were cured and discharged without any sequelae. Follow-up results revealed that none was reinfected by SFTSV. One of them had a car accident, one had a lung tumor, and one had a stroke; the rest of the patients still lived in the original place (tick epidemic area). Twelve patients reported that there were SFTS cases in their neighbors; five patients confirmed that they were bitten again by ticks, but there was no reinfection of SFTS among these patients.

### Dynamic Changes of IgM and Severe Fever With Thrombocytopenia Syndrome Virus Levels in Severe Fever With Thrombocytopenia Syndrome Patients

**FIGURE 1:**
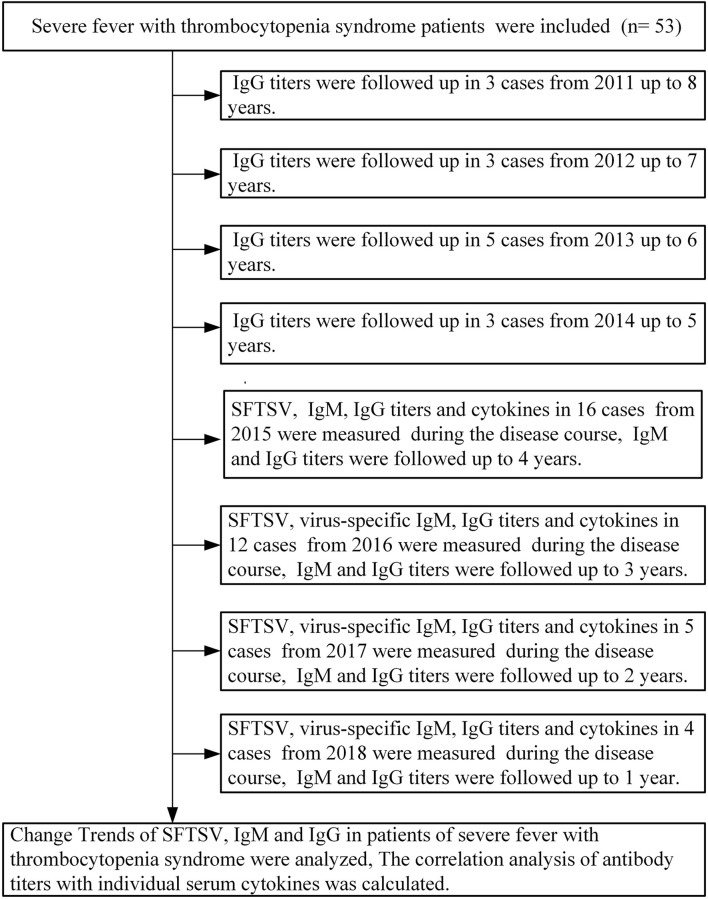
Diagram flow of patients with severe fever with thrombocytopenia syndrome included in this research. SFTSV, severe fever with thrombocytopenia syndrome virus; IgM, immunoglobulin M; IgG, immunoglobulin G.

Among 12 severe and 25 mild cases from 2015 to 2018, IgM antibodies and SFTSV were detected throughout the disease (Figure 1). The SFTSV load decreased gradually and cleared within 30 days from disease onset in all the 37 patients. Besides, we found SFTSV turned negative within 10 days from disease onset in two mild cases. The average time of SFTSV duration in the serum was 22.45 ± 7.6 days from onset. All the patients entered the convalescent stage at the second or third week of the disease course; they recovered within 1 month and the SFTSV disappeared.

**FIGURE 2:**
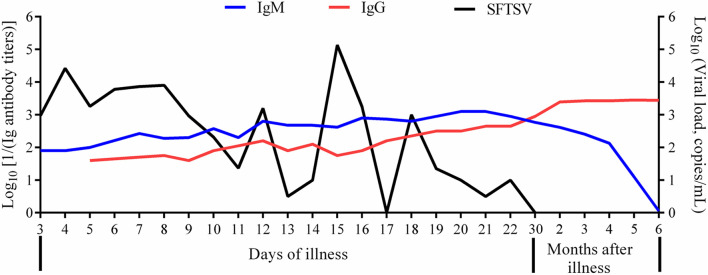
Change trends of SFTSV, IgM, and IgG in patients of severe fever with thrombocytopenia syndrome. SFTSV, severe fever with thrombocytopenia syndrome virus; IgM, immunoglobulin M; IgG, immunoglobulin G. The viral load was in the format of Log^10^ viral RNA copies/ml. The IgM titers were in the format of Log10 [1/(IgM antibody titers)]. The IgG titers were in the format of Log10 [1/(IgG antibody titers)].

As early as on the third day from the onset, IgM antibody positive seroconversion was detected in one patient. Nine patients produced IgM antibodies on the first week from onset, 19 cases produced IgM on the second week from onset, and 9 patients produced IgM on the third week. The average time of IgM antibody seroconversion was 10.7 ± 3.6 days from onset. The IgM titers that occurred firstly were between 1:80 and 1:640, then they increased gradually and attained higher peaking measurement up to 1:1,280 within the first 2 months after the disease among the patients. Three months after recovery, the IgM levels decreased gradually and could not be detected 6 months later in all the patients (Figure 2).

In this research, the correlation analyses showed that the higher the SFTSV load, the later the time of the IgM positive seroconversion; they had a positive correlation (Table 1). We also found that the time of IgM positive seroconversion was later in severe cases than that in mild cases (14.9 ± 0.6 vs. 8.8 ± 0.4 days, *p* < 0.0001). The data showed that the higher the levels of lactate dehydrogenase (LDH), creatine kinase (CK), and aspartate aminotransferase (AST), the later the IgM appeared (Table 1). Positive correlations of the time of IgM seroconversion with the fever time and hospitalization duration in SFTS patients during the disease course were also found (Table 1).

**TABLE 1:** Correlation matrix of IgM and production time with parameters about condition of patients with severe fever with thrombocytopenia syndrome.

**Parameters**	**SFTSV load^*a*^**		**LDH**		**CK**		**AST**		**Fever time**		**Hospitalization time**	
	***r* value**	***p*-value**	***r* value**	***p*-value**	***r* value**	***p*-value**	***r* value**	***p*-value**	***r* value**	***p*-value**	***r* value**	***p*-value**
IgM production time from onset (days)	0.75	<0.0001	0.408	0.015	0.398	0.018	0.372	0.03	0.706	<0.0001	0.73	<0.0001
IgG production time from onset (days)	0.546	0.002	0.502	0.004	0.533	0.002	0.464	0.009	0.7362	<0.0001	0.87	<0.0001

*SFTSV, severe fever with thrombocytopenia syndrome virus; LDH, lactate dehydrogenase; CK, creatine kinase; AST, aspartate aminotransferase; IgM, immunoglobulin M; IgG, immunoglobulin G.*

*^*a*^The viral load (in the format of Log^10^ viral RNA copies/mL) was subjected to the correlation analysis with the production time of IgM and IgG from onset (days).*

*For each correlation analysis, the respective correlation coefficient (r value) and P value of significance (shown in brackets) are presented, and P < 0.05 is considered significant.*

### IgG Antibody Levels in Severe Fever With Thrombocytopenia Syndrome Patients

Among 37 patients from 2015 to 2018, IgG antibodies were detected throughout the disease. IgG antibodies were produced firstly from the 5th to 30th days from the onset in SFTS patients; the average time of IgG antibody seroconversion was 17.3 ± 6.3 days from onset. IgG positive seroconversion occurred later in severe cases than in mild cases (23 ± 1.4 vs. 14.3 ± 1.0 days, *p* < 0.0001), but IgG titer levels were higher in severe cases. The correlation analyses also showed positive correlations of the levels of LDH, CK, and AST with the time during which IgG seroconversion occurred. The correlation analyses showed the later IgG was produced, the longer the fever time and hospitalization duration (Table 1).

**FIGURE 3:**
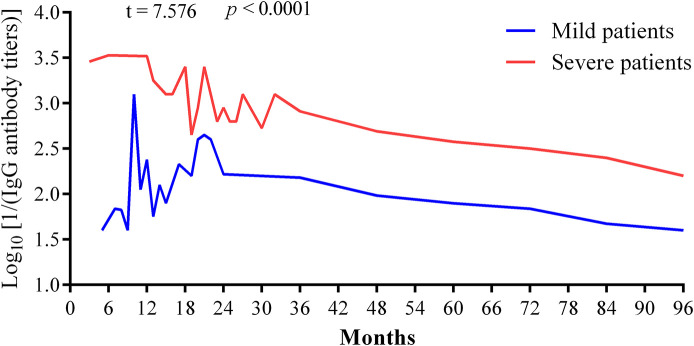
Change trends of IgG antibodies were compared among mild and severe cases of patients with severe fever with thrombocytopenia syndrome. The IgG titers were in the format of Log10 [1/(IgG antibody titers)]. Between the two groups of mild and severe cases, comparison of Log10 [1/(IgG antibody titers)] was calculated by unpaired *t*-test or non-parametric tests. A two-sided *p*-value < 0.05 was considered statistically significant.

Through follow-up among all the patients, IgG antibody levels increased gradually after seroconversion and got to the peak levels with titers from 1:1,280 [3.1 as in the format of Log10 (1280)] to 1:5120 [3.7 as in the format of Log10 (5120)] at the time of 6 months recovery of SFTS patients; the peak levels of IgG was maintained for about 1 year (Figure 1). Our results showed that IgG titers decreased gradually from the second year, which kept a more slow downward trend in severe cases than mild cases as in Figure 3 (2.953 ± 0.076 vs. 2.084 ± 0.084, *p* < 0.0001). The IgG antibodies can maintain up to 1:2560 [3.4 as in the format of Log10 (2560)] titers within 3 years, up to 1:1280 [3.1 as in the format of Log10 (1280)] titers on the fifth year, and up to 1:640 [2.8 as in the format of Log10 (640)] titers on the sixth year. It is noteworthy that one mild case in 2012 lost IgG antibody 7 years later. There are three SFTS cases in 2011 which still had positive IgG antibodies with titers from 1:80–1:160 (1.9–2.2) after 8 years.

Among the 37 patients, IgG levels (in the format of Log10 [1/(IgG antibody titers)]) were 3.47 ± 0.18; the median and interquartile range of IL-6, TNF-α, IL-10, and IL-8 among the patients was 76 (18–118), 70 (43–86), 95 (79–143), and 45 (31–119) pg/ml in the acute phase of the disease, respectively (Supplementary Table 1). The correlation analysis showed that the higher levels of IL-6, TNF-α, IL-10, and IL-8, the higher the titers of the IgG antibody (Table 2); a strong inflammatory response contributes to the persistent duration of IgG antibodies.

**TABLE 2:** Correlation matrix of IgG titers with inflammatory factors in patients with severe fever with thrombocytopenia syndrome.

**Parameters**	**IL-6**		**TNF-α**		**IL-10**		**IL-8**	
	***r*-value**	***p*-value**	***r*-value**	***p*-value**	***r* value**	***p*-value**	***r*-value**	***p*-value**
IgG titers^*a*^ (2 months later)	0.8319	<0.0001	0.6309	<0.0001	0.5368	0.0004	0.603	<0.0001
IgG titers (3 months later)	0.8461	<0.0001	0.6334	<0.0001	0.5218	0.0006	0.579	<0.0001
IgG titers (6 months later)	0.8087	<0.0001	0.5996	<0.0001	0.452	0.0034	0.555	0.0002
IgG titers (9 months later)	0.6324	<0.0001	0.4344	0.0036	0.4174	0.0074	0.452	0.0023
IgG titers (12 months later)	0.8044	<0.0001	0.6194	<0.0001	0.5074	0.0008	0.587	<0.0001
IgG titers (24 months later)	0.8173	<0.0001	0.6303	<0.0001	0.5281	0.0003	0.583	<0.0001
IgG titers (36 months later)	0.7004	<0.0001	0.4773	0.0028	0.5241	0.0009	0.432	0.0075
IgG titers (48 months later)	0.6937	0.0001	0.48	0.0152	0.6478	0.0005	0.492	0.0125

*IgG, immunoglobulin G; IL-6, interleukin-6; TNF-α, tumor necrosis factor-alpha; IL-10, interleukin-10; IL-8, interleukin-8.*

*^a^IgG titers (in the format of Log 2 IgG titer) were subjected to the correlation analysis with the levels of inflammatory factors.*

*For each correlation analysis, respective correlation coefficient (r value) and P-value of significance (shown in brackets) are presented, and P < 0.05 is considered as significant.*

## Discussion

SFTS was an emerging infectious disease with high mortality; so far, there is no effective treatment and vaccine against SFTSV. To our knowledge, there has been no report about how long the SFTSV specific antibody can be maintained in infected patients and whether the infected patients would be reinfected after recovery. In this research, we firstly indicated the contribution of the humoral immune response to SFTSV specific antibodies. Based on an 8-year follow-up study, we revealed that SFTSV specific IgG antibodies can be maintained for more than 8 years and show a downward trend, and severe SFTS patients can maintain higher levels of IgG antibody over a longer period.

Besides, there was no reinfection occurrence in all the SFTS patients even if some cases still lived in the tick epidemic area or in cases of re-exposure to SFTSV. This information might determine the important role of developing a vaccine against SFTSV. Our results also revealed that immunity induced by SFTSV contributes to IgM/IgG antibody seroconversion. The IgM antibody has value in the early diagnosis of SFTS. The long-term existence of IgG antibodies might have effective value to prevent SFTSV reinfection. Those rehabilitated cases with higher convalescence antibody levels might be considered to contribute plasma to patients as an option in therapy for SFTS.

The immune response plays an important role in the prognosis of viral infectious diseases (Sun et al., 2012a; Zhao et al., 2016; Hu et al., 2018). A moderate inflammatory response is protective and essential for resistance to infection; the dysregulated inflammatory response can be detrimental. An adaptive immune response also plays an important role in antibody production against virus infection. When severe or unbalanced immune system damage is caused by SFTSV in patients, the patients lose the ability of self-regulation, adaptive immunity would be prolonged or cannot be obtained, and effective antibodies cannot be produced; this may indicate a poor prognosis in critically ill SFTS patients. A moderate inflammatory response may be beneficial for production and duration of IgG antibodies. In our study, severe but not fatal illness was beneficial for the seroconversion and long-term maintenance of IgG antibody.

In this study, all the patients followed up had a better prognosis. During the SFTS course, when the SFTSV load in the patients was decreasing, the SFTSV specific IgM was produced and increased rapidly, but they disappeared after no more than 6 months, which was similar to a previous study (Lu et al., 2015). Like a previous report (Huang et al., 2019), our research also revealed that when the SFTSV turned negative in the early stage of the disease, IgM antibody had been produced in some mild cases; only based on SFTSV, it would miss the diagnosis. The average time of IgM positive seroconversion was on the 10th day from the onset which was similar to previous reports (Lu et al., 2015; Chang et al., 2018). Due to the existence of negative SFTSV RNA but positive SFTSV-specific IgM antibody in some patients, IgM antibody has the same early diagnostic value as SFTSV. We concluded that the IgM antibody has the same early diagnostic value as SFTSV. Besides, IgM antibody was found to be produced earlier in mild cases than severe cases, which suggested the important value of IgM to evaluate the prognosis of SFTS patients.

When patients infected by the virus recovered, specific IgG antibodies against the virus can be produced. IgG-specific antibodies produced by different viruses in the body can be maintained for different times ranging from a few months to a few decades. A previous study has estimated that IgG antibodies would become negative at 44 months after disease onset. However, our research revealed that IgG antibody can last for more than 8 years though they showed a downward trend in the body. To our knowledge, this is the longest follow-up study at present. One previous 4-year follow-up study has reported that antibodies against SFTSV existed 4 years after disease onset (Huang et al., 2016). Our research indicated that patients with SFTS who survived could produce long-lasting antibodies against SFTSV, but these did not exist for life. We reported one mild case who lost IgG antibody 7 years after the disease onset here.

What is noteworthy is that in this research none of the patients who survived were reinfected by SFTSV again. This information might implicate that patients with SFTS had neutralizing antibodies against SFTSV, which contribute to avoid reinfection. Further research can focus on the point. To our knowledge, we firstly showed positive correlations of the levels of inflammatory factors such as IL-6, TNF-α, IL-10, and IL-8 and the titers of IgG antibodies that were maintained. The initial seropositive time of IgG was later in severe cases than in mild cases; IgG can maintain higher titers in severe cases. We also firstly pointed out positive correlations between the initially seropositive time of IgG and the levels of LDH, CK, and ALT; the fever time; and the hospitalization of patients. However, the correlations of IgG seropositive time with levels of LDH, CK, and AST were not strongly positive; it may be related to the limited samples. Above all, we determined that moderate immune response in severe SFTS patients was beneficial for the seroconversion and long-term maintenance of IgG antibody; a strong inflammatory response can maintain the persistence of IgG antibody.

The limitations of our study include the relatively small number of patients studied, and SFTSV in the ticks collected from the patient’s body were not tested. Besides, case-fatality rates of SFTS varied between 2.5 and 33.3% in China. Our research focused on the survivors of SFTS; the correlation of IgM and IgG seroconversion time prognosis in fatal cases would need to be researched in our future study.

In summary, our study revealed negative conversions of SFTSV and SFTSV-specific IgM antibody were within 1 and 6 months from onset, respectively. We also found that the immune response elicited by SFTSV is beneficial to the production of SFTSV-specific IgG antibodies. Through an 8-year follow-up study, it was found that IgG antibodies could exist in the human body for a long time and effected protective immunity against SFTSV. It could be concluded that IgM has an important value in early diagnosis, and IgG has an important evaluation value of SFTS prognosis.

## Data Availability Statement

The original contributions presented in the study are included in the article/Supplementary Material, further inquiries can be directed to the corresponding author/s.

## Ethics Statement

The studies involving human participants were reviewed and approved by Ethics Committee of The First Affiliated Hospital of Anhui Medical University. The patients/participants provided their written informed consent to participate in this study.

## Author Contributions

LH and JbL performed conception and design of the study. LH, QK, YL, JjL, TB, XM, YY, and JbL performed experiments and collected and analyzed the data. LH, QK, YY, and JbL analyzed the data, wrote the manuscript, and revised the discussion. All authors contributed to the article and approved the submitted version.

## Conflict of Interest

The authors declare that the research was conducted in the absence of any commercial or financial relationships that could be construed as a potential conflict of interest.

## Publisher’s Note

All claims expressed in this article are solely those of the authors and do not necessarily represent those of their affiliated organizations, or those of the publisher, the editors and the reviewers. Any product that may be evaluated in this article, or claim that may be made by its manufacturer, is not guaranteed or endorsed by the publisher.
