# Supplementary material for: Time Course of Severe Fever With Thrombocytopenia Syndrome Virus and Antibodies in Patients by Long-Term Follow-Up Study, China
Source: Front Microbiol. 2021 Oct 12;12:744037. doi: 10.3389/fmicb.2021.744037 (PMC8546325; doi:10.3389/fmicb.2021.744037)
Supplement: Supplementary file 1 [file Data_Sheet_1.DOCX]

**Time Course of Severe Fever with Thrombocytopenia Syndrome Virus and Antibodies in Patients by Long-term Follow-up Study, China**

Lifen Hu^1, 3^, Qinxiang Kong^1, 2^, Yanyan Liu^1, 3^, Jiajia Li^3^, Tingting Bian^1^, Xuejiao Ma^1^, Ying Ye^1^*, Jiabin Li^1, 2, 3^*

^1^Department of Infectious Diseases, the First Affiliated Hospital of Anhui Medical University, Hefei, Anhui, China;

^2^Department of Infectious Diseases, Chaohu Hospital of Anhui Medical University, Hefei, Anhui, China.

^3^Anhui Center for Surveillance of Bacterial Resistance, Hefei, Anhui, China.

* Lifen Hu and Qinxiang Kong contributed equally to this work.

***Corresponding author:** Jiabin Li and Ying Ye

Address for Jiabin Li: Department of Infectious Diseases, the First Affiliated Hospital and Chaohu Hospital of Anhui Medical University, Jixi road 218, Hefei, Anhui, China

Tel: +86-551-62922713, Fax: +86-551-62922281, E-mail: lijiabin@ahmu.edu.cn

Address for Ying Ye: Department of Infectious Diseases, the First Affiliated Hospital of Anhui Medical University, Jixi Road 218, Hefei, Anhui, China

Tel: +86-551-62922713, Fax: +86-551-62922281, E-mail: yeying2@139.com

**Table.** The levels of immunoglobulin G antibody and inflammatory factors in patients with severe fever with thrombocytopenia syndrome

| Index | IgG levels (in the format of Log10 [1/(Ig G antibody titers)]) | | | | | | | |
| --- | --- | --- | --- | --- | --- | --- | --- | --- |
|  | 2 months(n=37) | 3 months (n=37) | 6 months(n=37) | 9 months(n=37) | 12 months(n=37) | 24 months(n=33) | 36 months(n=28) | 48 months(n=16) |
|  | 3.47 ± 0.18 | 3.48 ± 0.17 | 3.48 ± 0.16 | 3.36 ± 0.19 | 3.22 ± 0.15 | 3.14 ± 0.17 | 2.98 ± 0.18 | 2.85 ± 0.19 |
| IL-6,(pg/mL) | 76 (18-118) | 76 (18-118) | 76 (18-118) | 76 (18-118) | 76 (18-118) | 74(44, 147) | 76(44, 217) | 65(38, 126) |
| TNF-α, (pg/mL) | 70(43-86) | 70(43-86) | 70(43-86) | 70(43-86) | 70(43-86) | 65(34, 77) | 55(31, 79) | 50(28, 72) |
| IL-10, (pg/mL) | 95(79-143) | 95(79-143) | 95(79-143) | 95(79-143) | 95(79-143) | 100(84, 204) | 109(87, 265) | 105(87, 234) |
| IL-8, (pg/mL) | 45(31, 119) | 45(31, 119) | 45(31, 119) | 45(31, 119) | 45(31, 119) | 43(20, 55) | 36(20, 58) | 36(20, 47) |

Data are presented as the mean ± standard deviation, and median (interquartile range).

Abbreviations: IgG, immunoglobulin G; IL-6, interleukin-6; TNF-α, tumor necrosis factor-alpha; IL-8, interleukin-8; IL-10, interleukin-10.
