# Supplementary figures and images for: Time Course of Severe Fever With Thrombocytopenia Syndrome Virus and Antibodies in Patients by Long-Term Follow-Up Study, China
Source: Front Microbiol. 2021 Oct 12;12:744037. doi: 10.3389/fmicb.2021.744037 (PMC8546325; doi:10.3389/fmicb.2021.744037)

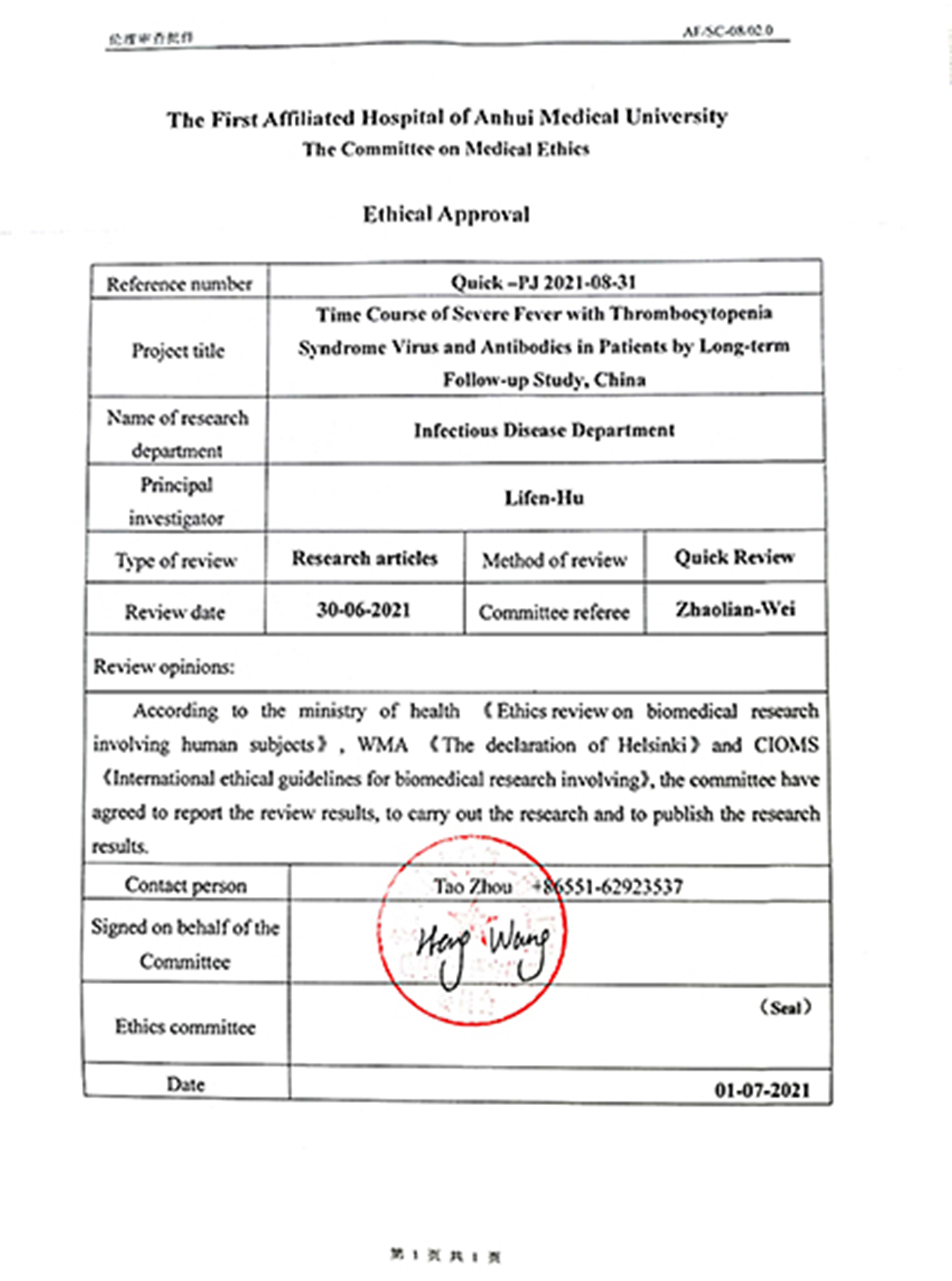

Supplement: Supplementary file 2 [file Image_1.JPEG]
